# Structural Insight Into Chitin Degradation and Thermostability of a Novel Endochitinase From the Glycoside Hydrolase Family 18

**DOI:** 10.3389/fmicb.2019.02457

**Published:** 2019-10-30

**Authors:** Yan-Jun Wang, Wen-Xin Jiang, Yi-Shuo Zhang, Hai-Yan Cao, Yi Zhang, Xiu-Lan Chen, Chun-Yang Li, Peng Wang, Yu-Zhong Zhang, Xiao-Yan Song, Ping-Yi Li

**Affiliations:** ^1^State Key Laboratory of Microbial Technology, Marine Biotechnology Research Center, Shandong University, Qingdao, China; ^2^College of Marine Life Sciences, Institute for Advanced Ocean Study, Ocean University of China, Qingdao, China; ^3^Laboratory for Marine Biology and Biotechnology, Pilot National Laboratory for Marine Science and Technology, Qingdao, China

**Keywords:** endochitinase, GH18 family, chitin degradation, substrate binding, thermostability

## Abstract

Bacterial endochitinases play important roles in environmental chitin degradation and have good applications. Although the structures of some endochitinases, most belonging to the glycoside hydrolase (GH) family 18 and thermostable, have been reported, the structural basis of these enzymes for chitin degradation still remain unclear due to the lack of functional confirmation, and the molecular mechanism for their thermostability is also unknown. Here, we characterized a GH18 endochitinase, Chi23, from marine bacterium *Pseudoalteromonas aurantia* DSM6057, and solved its structure. Chi23 is a thermostable enzyme that can non-processively hydrolyze crystalline and colloidal chitin. Chi23 contains only a catalytic domain that adopts a classical (β/α)_8_ TIM-barrel fold. Compared to other GH18 bacterial endochitinases, Chi23 lacks the chitin-binding domain and the β-hairpin subdomain, indicating that Chi23 has a novel structure. Based on structural analysis of Chi23 docked with (GlcNAc)_5_ and mutational analysis, the key catalytic residue (Glu117) and seven substrate-binding residues (Asn9, Gln157, Tyr189, Asn190, Asp229, Trp260, and Gln261) are revealed. Among these identified residues, Asn9, Asp229 and Gln261 are unique to Chi23, and their cumulative roles contribute to the activity of Chi23 against both crystalline and soluble chitin. Five substrate-binding residues (Tyr189, Asn190, Asp229, Trp260, and Gln261) are found to play important roles in maintaining the thermostability of Chi23. In particular, hydrogen bond networks involving Asp229 and Gln261 are formed to stabilize the protein structure of Chi23. Phylogenetic analysis indicated that Chi23 and its homologs represent a new group of GH18 endochitinases, which are widely distributed in bacteria. This study sheds light on the molecular mechanism of a GH18 endochitinase for chitin degradation.

## Introduction

Chitin, an insoluble linear polysaccharide of β-1,4 linked *N*-acetyl-D-glucosamine (GlcNAc), is the second most abundant biopolymer in nature after cellulose. In nature, chitin is organized in crystalline arrangements and constitutes the main structural component of arthropod exoskeletons, fungal cell walls and insect cuticles. Colloidal chitin is a kind of chitin comprising an amorphous part and a relatively crystalline part. Compared to crystalline chitin, colloidal chitin is easier to be degraded by chitinases. Chitinases, including endochitinases, exochitinases and *N*-acetylglucosaminases, can hydrolyze chitin into chitin oligosaccharides and/or monosaccharides, and are widely distributed in bacteria (Suginta et al., 2016), fungi (Takaya et al., 1998) and plants (Cavada et al., 2006), which play key roles in natural chitin degradation and recycling. Chitinases are also gaining increasing attention in medicine, agriculture, food industry and environmental management (Oyeleye and Normi, 2018). Endochitinases are attractive in chitin oligosaccharide preparation and other industrial applications due to their distinct action mode from exochitinases and *N*-acetylglucosaminases. However, endochitinases reported to be active on crystalline chitin are still limited, which therefore, need to be further explored.

Bacterial chitinases are distributed in glycoside hydrolase (GH) families 3, 5, 18, 19, 20, 23, 48, 84, and 116. Among chitinases, those from the GH family 18 (GH18) are most extensively studied due to their large amount in nature and efficient degradation of crystalline chitin. The catalytic domains (CaD) of GH18 chitinases adopt the classical (β/α)_8_ TIM-barrel fold with a conserved catalytic DxDxE motif (Perrakis et al., 1994; Matsumoto et al., 1999). All GH18 chitinases are retaining enzymes, and their catalysis depends on the double displacement substrate-assisted mechanism (van Aalten et al., 2001; Oyeleye and Normi, 2018). Until now, a considerable amount of GH18 chitinases are reported to be thermostable at high temperatures, making them good candidates for industrial application (Wen et al., 2002; Oku and Ishikawa, 2006). However, the molecular basis for their thermostability is largely unknown.

Based on sequence similarity, GH18 bacterial chitinases are grouped into three subfamilies, A, B, and C (Suzuki et al., 1999). Most characterized GH18 bacterial chitinases belong to the subfamily A. Chitinases of this subfamily are commonly processive exochitinases with a deep substrate cleft owing to the insertion of a small chitin insertion domain (CID) into their CaD domains (Perrakis et al., 1994; Matsumoto et al., 1999; Houston et al., 2002; Pantoom et al., 2011; Chen et al., 2018). Processive chitinases play an important role in efficient degradation of crystalline chitin, and the underlying degradation mechanisms have been studied. Structural analysis has revealed the importance of aromatic residues in the CaD and CID for enzyme activity and processivity (Watanabe et al., 2003; Suginta et al., 2007; Zakariassen et al., 2009; Li and Greene, 2010). Tryptophans in the chitin-binding domain (CBD) and other additional domains are also found to have roles in crystalline chitin hydrolysis (Uchiyama et al., 2001; Katouno et al., 2004). Recently, by using single-molecule imaging, the processive movements of *Serratia marcescens* chitinase A (*Sm*ChiA) on crystalline chitin were directly observed (Nakamura et al., 2018a), and its rate constants and productive binding ratio were revealed (Nakamura et al., 2018b).

In contrast to the extensive studies on the chitinases from subfamily A, only a limited number of bacterial chitinases from subfamily B are characterized, including ChiNCTU2 from *Bacillus cereus* NCTU2 (Wen et al., 2002), *Mm*Chi60 from *Moritella marina* (Stefanidi and Vorgias, 2008), *Cj*Chi18C from *Cellvibrio japonicus* (Monge et al., 2018), *Sm*ChiC from *Serratia marcescens* (Suzuki et al., 1999), *Sp*ChiC from *Serratia proteamaculans* (Purushotham et al., 2012) and *Ss*Chi18B from *Streptomyces* sp. F-3 (Sun et al., 2019). Different from subfamily A chitinases that are processive exochitinases, all reported subfamily B chitinases but ChiNCTU2 are modular endochitinases containing a CaD and one or more CBDs, which non-processively hydrolyze crystalline and colloidal chitin. ChiNCTU2 is an exochitinase containing only a CaD, which can hydrolyze colloidal chitin but not crystalline chitin (Wen et al., 2002). Among these enzymes, only the structures of ChiNCTU2 (Hsieh et al., 2010), *Mm*Chi60 (Malecki et al., 2013) and *Sm*ChiC (Payne et al., 2012) are reported. All the catalytic domains of the subfamily B chitinases lack a CID and have a shallow substrate-binding cleft (Hurtado-Guerrero and van Aalten, 2007; Hsieh et al., 2010; Payne et al., 2012; Malecki et al., 2013). Structural analyses have revealed that the subfamily B chitinases harbor fewer aromatic residues than those of subfamily A (Hurtado-Guerrero and van Aalten, 2007; Hsieh et al., 2010; Payne et al., 2012; Malecki et al., 2013). Although potential residues involved in substrate binding and catalysis for the subfamily B chitinases have been suggested based on the limited structures, their contributions to chitin degradation, especially for crystalline chitin, are still unclear.

Thermostable endochitinases active on crystalline chitin have good industrial and biotechnological potentials. However, reports on such enzymes are still limited. In this study, we characterized a thermostable endochitinase (Chi23) that can hydrolyze both crystalline and colloidal chitin. Chi23, from a marine bacterium *Pseudoalteromonas aurantia* DSM6057, is a member of the subfamily B of the GH18 family. However, different from other modular endochitinases of the subfamily B, Chi23 is a single-domain enzyme containing only a catalytic domain. We solved the crystal structure of the wild-type Chi23 and modeled its structure with (GlcNAc)_5_. Based on structural and mutational analyses, the key residues of Chi23 involved in substrate binding and catalysis were revealed, and its structural basis for chitin degradation and for high thermostability was also probed.

## Materials and Methods

### Sequence Analysis of Chi23

MUSCLE was used to perform multiple sequence alignment (Edgar, 2004). Software MEGA 7.0 was used for phylogenetic analysis (Kumar et al., 2016). SignalP 4.1 (Petersen et al., 2011) was used to identify the potential signal peptide sequence.

### Gene Cloning and Mutagenesis

*Pseudoalteromonas aurantia* DSM6057 was obtained from the DSMZ. The *chi23* gene was amplified from the genomic DNA of *P. aurantia* DSM6057 via PCR using gene-specific primers (Table 1). The amplified fragment was ligated into the vector pET22b to construct the recombinant plasmid pET22b-*chi23*. Site-directed point mutants were created by the QuikChange mutagenesis method (Xia et al., 2014) with primers containing mutations (Table 1) and with plasmid pET22b-*chi23* as the template. All the recombinant plasmids were verified by sequencing.

**TABLE 1:** Primers used in this study.

**Gene product**	**Primer**	**Sequence (5′-3′)^a^**
Chi23	Chi23-F Chi23-R	AAGAAGGAGATATACATATGTCTAAAACCATTACCTATTATAACTCG (*Nde*I) TGGTGGTGGTGGTGCTCGAGGCTATTCAGCGATTGTGCAA (*Xho*I)
N9A	N9A-F N9A-R	TATTATGCCTCGGGCGCGGTCCCGCTCAT CCCGAGGCATAATAGGTAATGGTTTTAGACAT
G79A	G79A-F G79A-R	TCATTTGGTGGCGCCACCATGGGTTCCAACGCTT TGGCGCCACCAAATGAGATCAATACTTTCTGCCCCTTGTG
T80A	T80A-F T80A-R	CGCCATGGGTTCCAACGCTTATCGTTCGTTGTCA TTGGAACCCATGGCGCCGCCACCAAATGAGATC
D115A	D115A-F D115A-R	ATATAGCTTATGAAGATACGGCCGCGTTCACTGGTCAAG GCCGTATCTTCATAAGCTATATCCACGCCATCTAACTGAT
E117A	E117A-F E117A-R	TATAGATTATGCAGATACGGCCGCGTTCACTGGTCAAG GCCGTATCTGCATAATCTATATCCACGCCATCTAACTGAT
Q157A	Q157A-F Q157A-R	ATTTCTCATGCACCTGCACCTCCTTATTTGGAGCAAGGC TGCAGGTGCATGAGAAATGATGTAGTCAGGGCTCGG
Y189A	Y189A-F Y189A-R	TTAAATGTGCAGTTTGCCAACAACCCGCCATGG GGCAAACTGCACATTTAACCAGTCAATCTCTTGTCCC
N190A	N190A-F N190A-R	TGTGCAGTTTTACGCCAACCCGCCATGGAGTGCT TTGGCGTAAAACTGCACATTTAACCAGTCAATCTCTTGTCCCA
D229A	D229A-F D229A-R	TGTCACGCAGAACGCTGCGGGTTCTGGGTATATGC AGCGTTCTGCGTGACAGGAAAGCCAGCAATAACCTTCTC
W260A	W260A-F W260A-R	ATTATGAATGCGCAGTTCTCAAGTGACCACAATGGT AACTGCGCATTCATAATGCCGCCAAGGCTAGAT
Q261A	Q261A-F Q261A-R	ATTATGAATTGGGCGTTCTCAAGTGACCACAATGGTGATT AGAACGCCCAATTCATAATGCCGCCAAGGCTAGAT

*^*a*^The cloning sites used are underlined, and the restriction enzymes are indicated in parentheses.*

### Protein Expression, Purification and Zymogram Analysis

The wild-type Chi23 protein and all mutants were expressed in *Escherichia coli* BL21 (DE3) cells and induced by the addition of 1 mM isopropyl-β-D-thiogalactopyranoside at 20°C and 110 rpm for 20 h. Cells were collected and disrupted by pressure in a binding buffer (50 mM Tris-HCl, 100 mM NaCl, pH 8.0) containing 5 mM imidazole. The crude extract was loaded onto Ni-NTA agarose resin (Qiagen, United States), washed with 15 mM imidazole in the binding buffer, and eluted with 350 mM imidazole in the binding buffer. Recombinant His-tagged proteins were further purified by gel filtration chromatography on a Superdex-200 column (GE Healthcare, Sweden) equilibrated with 10 mM Tris-HCl buffer (pH 8.0) containing 100 mM NaCl. The eluted enzyme fractions were checked on SDS-PAGE (12.5%). Zymogram analysis was also performed to confirm the target protein. Chitinase activity of the purified protein was detected on gels by using the fluorescent substrate 4-methylumbelliferyl-β-D-*N,N’*-diacetylchitobiose [4-MU-(GlcNAc)_2_] (Sigma, United States) (Barboza-Corona et al., 1999; Nagpure et al., 2014). Protein concentrations were determined by Pierce BCA Protein Assay Kit (Thermo Scientific, United States).

### Colloidal Chitin Preparation

Colloidal chitin was prepared as previously described (Roberts and Selitrennikoff, 1988) with some modification. Briefly, 10 g of powdered chitin (Carbosynth, China) was dissolved in cold concentrated HCl (200 mL) and stirred at 180 rpm and 10°C for 2 h. 2.5 L of cold deionized water was added to the hydrolysate with continuous stirring for 10 min and the resultant chitin suspension was centrifugated at 9,000 rpm and 4°C for 10 min. The chitin pellets were washed repeatedly with cold deionized water until the pH of the filtrate was about 5.0, and then dissolved in deionized water to the concentration of 50 mg/mL.

### Enzymatic Activity Assays

Chitinase activity was determined by measuring the production of reducing sugars from colloidal or crystalline chitin. The standard reaction system (220 μL) contained 50 mM sodium acetate buffer (pH 5.0), 0.8–15 μM enzyme and 10 mg/mL colloidal chitin, which was replaced by 60 mg/mL crystalline chitin in a final volume of 1 mL when crystalline chitin was used as substrate. After incubation at 60°C for 15 min (for colloidal chitin substrate) or 20 min (for crystalline chitin substrate), the reaction was terminated by the addition of dinitrosalicylic acid (DNS) into the mixture. The amount of reducing sugars released into the mixture was determined with GlcNAc as the standard using the DNS method (Miller, 1959). One unit of enzyme activity is defined as the amount of enzyme required to release 1 μmol of reducing sugars per min.

### Biochemical Characterization

Substrate specificity assays of Chi23 were carried out with crystalline chitin, colloidal chitin, glycol chitosan, chitosan and avicel. The optimum temperature (*T*_*opt*_) for Chi23 and its mutants was measured at temperatures ranging from 0 to 90°C at pH 5.0. For thermostability assay, Chi23 was preincubated at temperatures ranging from 60°C to 80°C for different periods of time, and the residual activity was measured at 60°C. The optimum pH of Chi23 was determined at 60°C in the Britton-Robinson buffer ranging from pH 2.0 to 10.0. For pH stability assay, the enzyme was preincubated in buffers with a pH range of 2.0–13.0 at 0°C for 1 h, and then the residual activity was measured at pH 5.0 and 60°C. The effect of NaCl on Chi23 activity was determined at NaCl concentrations ranging from 0 to 4.0 M. For halotolerance assay, the enzyme was incubated at 0°C for 1 h in buffers containing NaCl ranging from 0 to 4.5 M before the residual activity was measured at 60°C. The effects of selected metal ions on Chi23 activity were examined at pH 5.0 and 60°C in a final concentration of 1 or 10 mM.

Enzyme kinetics assays were carried out in McIlvaine’s buffer (pH 5.0) using 4-MU-(GlcNAc)_2_ at concentrations from 0.01 to 0.6 mM. Kinetic parameters were calculated by non-linear regression fit directly to the Michaelis–Menten equation using the Origin8 software. The overall secondary structures of wild-type Chi23 and its mutants were investigated at 25°C using a J-810 circular dichroism (CD) spectropolarimeter (Jasco, Japan). CD spectra were collected from 200 to 250 nm at a scanning rate of 200 nm/min with a path length of 0.1 cm. All proteins used were at a concentration of 9.8 μM in 10 mM Tris-HCl buffer (pH 8.0) containing 100 mM NaCl.

### Apparent Melting Temperature (*T*_*m*_)

Differential scanning calorimetry (DSC) measurements were carried out over a temperature range of 10 to 110°C at a scanning rate of 1°C/min using a MicroCal VP-DSC microcalorimeter (GE Healthcare, Sweden). The sample solutions contained approximately 29.5 μM enzyme dissolved in Tris-HCl buffer (10 mM Tris-HCl, 100 mM NaCl, pH 8.0), and the Tris-HCl buffer was used as reference solution for accuracy. Prior to loading, all solutions were filtered using 0.22 μm pore size membrane filter and degassed. All data were analyzed using Origin8 software and further processed by fitting to a non-2-state model.

### Analysis of Hydrolysis Products From Chitooligomers

Thin layer chromatography (TLC) analysis was carried out as described by Huang and Chen (2005) with some modification. Reaction mixture (60 μL) containing 3.3 mg/mL of chitooligomers (chitobiose-chitohexaose) and 32.8 nM of Chi23 was incubated at 60°C for 12 h. After boiling the mixtures for 15 min, the reaction products were spotted onto a silica gel plate and developed with *n*-butyl alcohol-acetic acid-water-ammonia (10:5:5:1, v/v/v/v) for 3 h. To visualize the hydrolysis products, the developed plate was sprayed using a diphenylamine-aniline-phosphate reagent (0.8 g diphenylamine, 40 mL acetone, 0.8 mL aniline and 4 mL of 85% phosphoric acid) and then heated at 105°C for 10 min.

Time course of (GlcNAc)_6_ hydrolysis by the wild-type Chi23 was also measured. Hydrolysis of (GlcNAc)_6_ by Chi23 was carried out at pH 5.0 and 60°C for 5–120 min and the concentrations of the enzyme and substrate used were 32.8 nM and 6.2 mg/mL, respectively. The resultant hydrolysis products were analyzed by gel filtration chromatography on a Superdex Peptide 10/300 GL column (GE Healthcare, Sweden) at a flow rate of 0.4 mL/min using 0.2 M ammonium hydrogen carbonate as the running buffer. Elution was monitored at 210 nm using a UV detector. LabSolutions software was used to online monitoring and data analysis.

### Crystallization, Data Collection, and Structure Determination

Wild-type Chi23 for crystallization was diluted to 5 mg/mL in 10 mM Tris-HCl (pH 8.0) containing 100 mM NaCl. Chi23 crystals grew at 18°C in the buffer containing 0.05 M KH_2_PO_4_ and 20% (w/v) PEG 8000. All the x-ray diffraction data were collected on the BL18U1 Beamline at Shanghai Synchrotron Radiation Facility using Area Detector Systems Corporation Quantum 315r. The initial diffraction data sets were processed by the HKL3000 program (Minor et al., 2006). The crystal structure of Chi23 was solved by molecular replacement using the PPL2 structure (PDB code 2GSJ) as the starting model. Subsequent refinement was performed using Coot (Emsley and Cowtan, 2004) and Phenix (Adams et al., 2002). All structure figures were generated using PyMOL software.

### Molecular Docking

Schrödinger software^1^ was used to conduct the Chi23 and (GlcNAc)_5_ docking. The crystal structure of Chi23 was first optimized by Protein Preparation Wizard to optimize the structure and minimize the energy to make it more stable. Then Schrödinger LigPrep software (LigPrep, Schrödinger, LLC, New York, NY, United States, 2019) was used to preprocess (GlcNAc)_5_ to obtain its low energy three dimensional conformers. Finally, (GlcNAc)_5_ was docked into the binding site of minimized Chi23 using the Glide (Friesner et al., 2006) with the standard precision scoring mode. In molecular docking, the candidate with the lowest binding energy was chosen.

## Results and Discussion

### Sequence Analysis of Chi23

**FIGURE 1:**
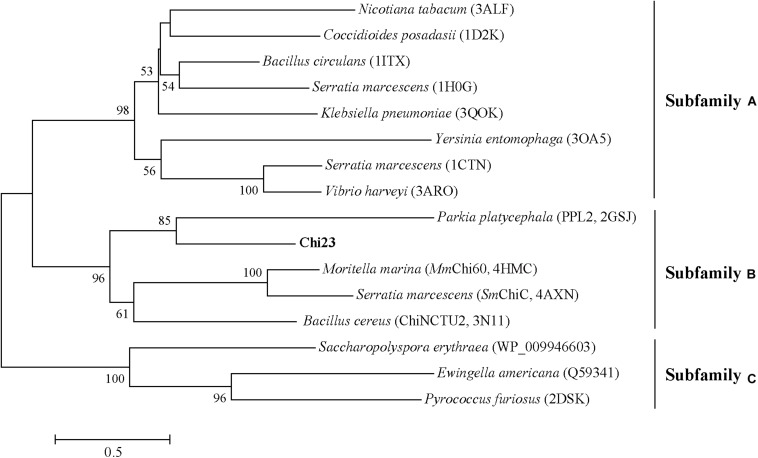
Phylogenetic analysis of Chi23 and other chitinases from the GH18 family. The unrooted phylogenetic tree was constructed by using the neighbor-joining method with a Jones–Taylor–Thornton (JTT) matrix-based model using 202 amino acid positions. Bootstrap analysis of 1,000 replicates was conducted, and values above 50% are shown.

**FIGURE 2:**
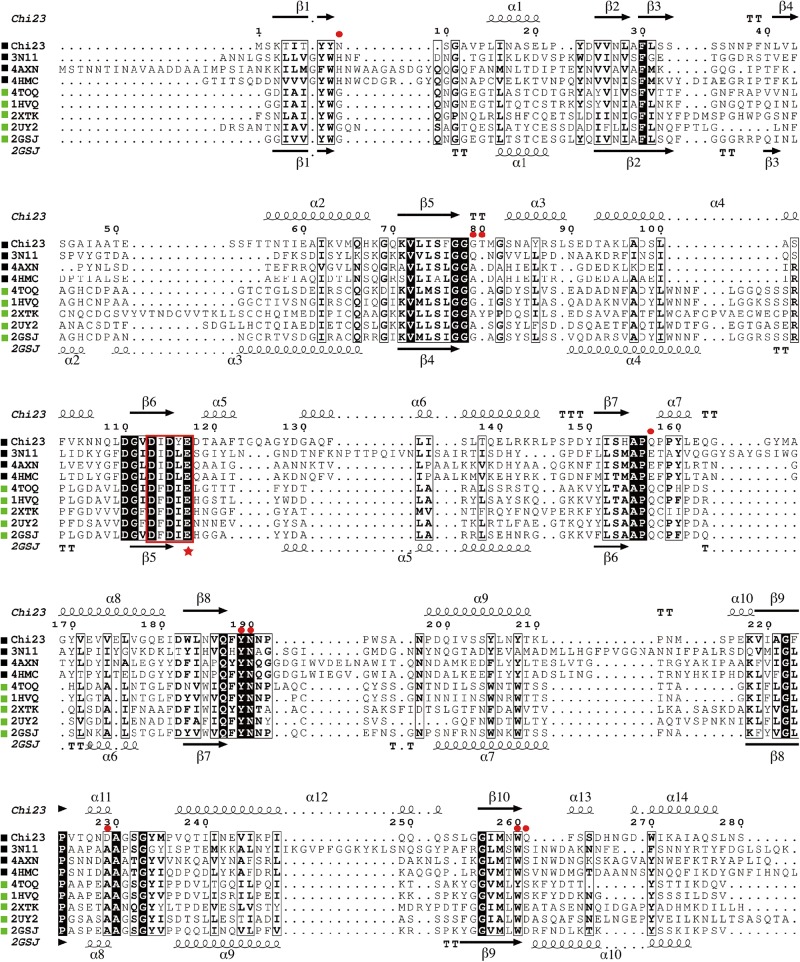
Multiple sequence alignment of Chi23 and other chitinases from the GH18 subfamily B. Using ESPript 3.0, secondary structures of Chi23 are shown above alignment and those of the endochitinase PPL2 (PDB code 2GSJ) from *Parkia platycephala* seeds under alignment. Helices are indicated by springs, strands by arrows, turns by TT letters. Identical residues are shown in white on a black background, and similar residues are in bold black. Chitinases from bacteria and eukaryotes are marked by black and green squares, respectively. The conserved DxDxE motif containing the catalytic Glu (marked by a star) is boxed. Selected residues of Chi23 for mutation are indicated by red circles.

Chitin utilization experiment indicated that the marine bacterium *P. aurantia* DSM6057 (Gauthier and Breittmayer, 1979) could use colloidal chitin as the sole carbon source, suggesting that this strain produces chitinases for chitin degradation. We then sequenced the genome of this strain to find out its chitinases. Based on gene annotation, a gene encoding a putative chitinase was identified from the genome sequence, which was named *chi23*. The Chi23 protein comprises 280 amino acid residues with a calculated molecular mass of 30.4 kDa, and no signal peptide sequence can be predicted in Chi23 sequence by SignalP 4.1. Among the characterized enzymes, Chi23 is most closely related to the GH18 endochitinase PPL2 from *Parkia platycephala* seeds (Cavada et al., 2006) with a low sequence identity of 30%. By blasting against the Conserved Domains Database (CDD), Chi23 was found to be a single-domain enzyme, containing only a GH18 catalytic domain. Phylogenetic analysis showed that Chi23 belongs to the subfamily B of the GH18 family (Figure 1). Multiple sequence alignment suggested that the key catalytic residue of Chi23 is Glu117, which is located in the typical DxDxE motif (Matsumoto et al., 1999) of the GH18 chitinases (Figure 2).

### Biochemical Characterization of Chi23

**FIGURE 3:**
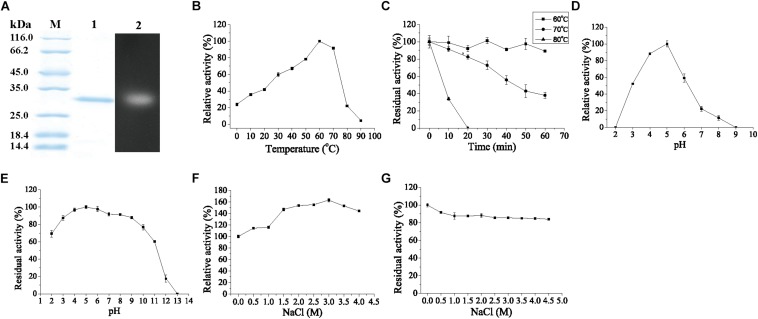
SDS-PAGE and zymogram analysis of purified Chi23 and Biochemical characterization of Chi23 using colloidal chitin as substrate. **(A)** SDS-PAGE and zymogram analysis of purified Chi23. Lane M, protein mass markers; lane 1, purified Chi23; lane 2, zymogram of Chi23. **(B)** Effect of temperature on the activity of Chi23. **(C)** Effect of temperature on the stability of Chi23. **(D)** Effect of pH on the activity of Chi23. **(E)** Effect of pH on the stability of Chi23. **(F)** Effect of NaCl on the activity of Chi23. **(G)** Effect of NaCl on the stability of Chi23. The graphs show data from triplicate experiments (means ± SD).

Chi23 was over-expressed in *E. coli* BL21 (DE3) and purified. SDS-PAGE analysis showed that the purified Chi23 displays an apparent molecular mass of approximately 30 kDa, accordant to that predicted from its sequence (30.4 kDa) (Figure 3A). Only one fluorescent band with an apparent molecular mass of 30 kDa was observed in zymogram analysis, indicating that the purified Chi23 is an active chitinase (Figure 3A). Recombinant Chi23 could hydrolyze both crystalline and colloidal chitin, but showed little activity toward glycol chitosan, chitosan or avicel (Table 2). The optimal temperature for Chi23 activity was 60°C (Figure 3B). Chi23 was stable at 60°C, and retained over 40% of its maximal activity after 1 h incubation at 70°C (Figure 3C), suggesting that Chi23 is a thermostable enzyme. Chi23 had the highest activity at pH 5.0 and showed good tolerance over a wide pH range, retaining over 60% activity at pH 2.0–11.0 (Figures 3D,E). Similar to *Mm*Chi60 (Stefanidi and Vorgias, 2008), basic solutions with pH values in a range of 8.0–10.0 had small impact on Chi23 stability, but severely decreased or fully abolished the activity of Chi23, suggesting that the protein structure of Chi23 is possibly maintained under basic conditions. Chi23 activity could be stimulated by 3.0 M NaCl by 1.6 folds (Figure 3F), consistent with its marine origin. Moreover, after 1 h incubation in 4.5 M NaCl, Chi23 still retained 84% activity (Figure 3G). Thus, Chi23 is a thermostable, halotolerant, and acidic chitinase. Like other GH18 subfamily B chitinases (Wen et al., 2002; Stefanidi and Vorgias, 2008), Chi23 activity was hardly affected by K^+^, Li^+^, Ba^2+^, Ca^2+^, Mg^2+^ or Ni^2+^ at 1–10 mM concentrations, but severely inhibited by Cu^2+^ at 10 mM concentration (Table 3). Chi23 activity was also strongly inhibited by Co^2+^, Fe^2+^, and Mn^2+^ at 10 mM concentration, which, however, had no effect on the activity of *Mm*Chi60 (Stefanidi and Vorgias, 2008).

**TABLE 2:** The substrate specificity of Chi23.

**Substrate**	**Specific activity (U/mg)**
Crystalline chitin	0.1 ± 0.01
Colloidal chitin	1.2 ± 0.02
Glycol chitosan	LD^a^
Chitosan	LD
Avicel	LD

*^*a*^LD indicates that the value was less than the limit of detection.*

**TABLE 3:** Effects of metal ions on Chi23 activity.

**Metal ions**	**Relative activity (%)**	
	**1 mM**	**10 mM**
None	100	100
K^+^	106.8 ± 1.6	100.1 ± 2.6
Li^+^	106.5 ± 1.5	122.1 ± 3.0
Ba^2+^	109.7 ± 3.5	105.2 ± 1.9
Ca^2+^	109.0 ± 1.3	104.2 ± 1.4
Co^2+^	57.0 ± 1.3	27.0 ± 0.6
Cu^2+^	40.8 ± 1.5	LD^a^
Fe^2+^	60.0 ± 1.7	10.9 ± 1.2
Mg^2+^	125.9 ± 1.1	106.3 ± 1.5
Mn^2+^	37.2 ± 2.7	3.7 ± 1.3
Ni^2+^	116.5 ± 2.8	104.6 ± 2.2
Zn^2+^	84.0 ± 4.0	57.0 ± 0.4

*^*a*^LD indicates that the value was less than the limit of detection.*

**FIGURE 4:**
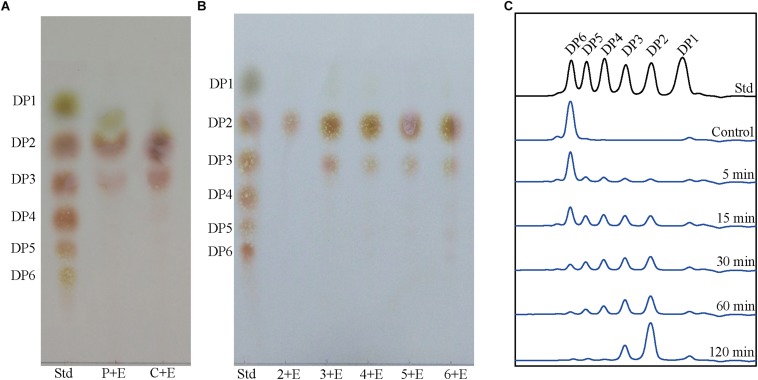
Hydrolysis of crystalline chitin, colloidal chitin and chitooligomers by the purified Chi23. **(A)** TLC showing the complete hydrolysis of crystalline chitin (powder) and colloidal chitin by Chi23. Lane Std, standard mix of (GlcNAc)_1__–__6_; lane P + E, hydrolysis of crystalline chitin; lane C + E, hydrolysis of colloidal chitin. **(B)** TLC showing the complete hydrolysis of chitooligomers (GlcNAc)_2__–__6_ by Chi23. Lane Std, standard mix of (GlcNAc)_1__–__6_; lanes 2 + E to 6 + E, hydrolysis of chitooligomers from (GlcNAc)_2_ to (GlcNAc)_6_, respectively. **(C)** Time course of (GlcNAc)_6_ hydrolysis by Chi23. Hydrolysis of (GlcNAc)_6_ was carried out at pH 5.0 and 60°C for 5–120 min and the concentrations of the enzyme and substrate used were 32.8 nM and 6.2 mg/mL, respectively.

TLC analysis showed that (GlcNAc)_2_ and (GlcNAc)_3_ were the predominant products when crystalline chitin, colloidal chitin and chitooligomers (chitotriose-chitohexaose) were hydrolyzed by Chi23 (Figures 4A,B). Chi23 could not hydrolyze (GlcNAc)_2_, and the products from (GlcNAc)_6_ degradation were not all (GlcNAc)_2_ (Figure 4B), suggesting that Chi23 functions as an endochitinase rather than an exochitinase. To reveal the action mode of Chi23, time course of (GlcNAc)_6_ hydrolysis by Chi23 was performed. Degradation products from (GlcNAc)_6_ by Chi23 were (GlcNAc)_2_, (GlcNAc)_3_ and (GlcNAc)_4_ at first, and the resulting (GlcNAc)_4_ was subsequently hydrolyzed into (GlcNAc)_2_ (Figure 4C), which further indicated that Chi23 is an endochitinase.

### Overall Structural Analysis of Chi23

**FIGURE 5:**
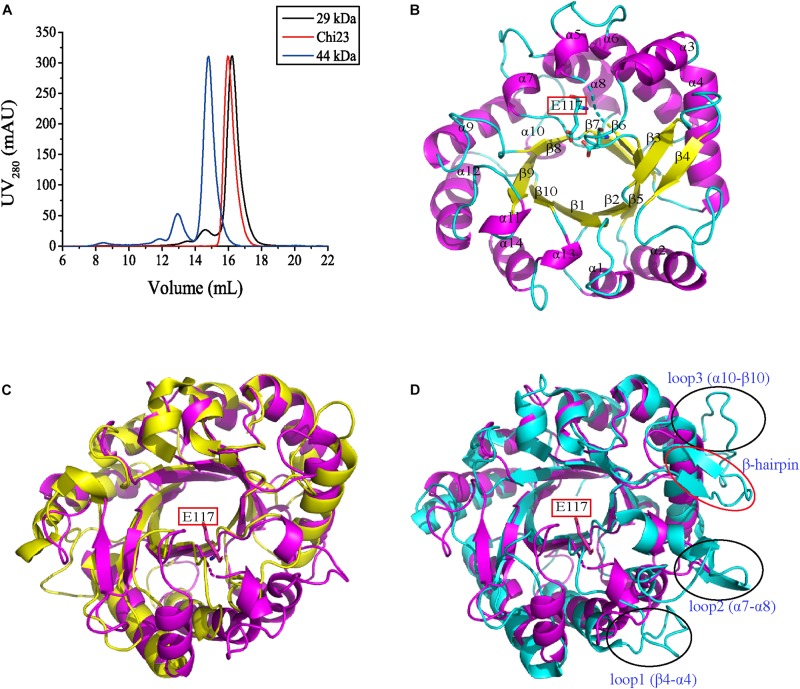
Structural analysis of Chi23. **(A)** Gel filtration analysis of recombinant Chi23 and markers. Chi23 monomer has a calculated molecular mass of 30.4 kDa. Two protein size markers are carbonic anhydrase (29 kDa) and ovalbumin (44 kDa). **(B)** The overall structure of Chi23. The key catalytic residue of Chi23 is shown in sticks. **(C)** Structural superimposition of Chi23 (magenta) and PPL2 (yellow). **(D)** Structural superimposition of Chi23 (magenta) and ChiNCTU2 (cyan). ChiNCTU2 has a β-hairpin subdomain (marked by red circle) and three long loops (marked by black circles) in structure, which are all absent in Chi23 and ChiNCTU2.

To ascertain the structural basis of Chi23 for chitin degradation, the crystal structure of Chi23 was solved at 1.80-Å resolution by molecular replacement using the PPL2 structure (30% sequence identity with Chi23, PDB code 2GSJ) (Cavada et al., 2006) as the starting model. The crystallographic and refinement statistics are summarized in Table 4. The crystal of Chi23 belongs to the *P*12_1_ space group with four molecules loosely packed per asymmetric unit. Gel filtration analysis showed that Chi23 presents as monomers in solution (Figure 5A). Consistent with sequence analysis, structural analysis also showed that Chi23 is a single-domain protein containing only a CaD, which is distinct from other endochitinases of the GH18 subfamily B that are all modular proteins (Payne et al., 2012; Malecki et al., 2013). Like the catalytic domains of the other chitinases from the GH18 subfamily B, the overall structure of Chi23 adopts the classical (β/α)_8_ TIM-barrel fold (Figure 5B), most closely resembling the structures of PPL2 (Cavada et al., 2006) and ChiNCTU2 (PDB code 3N11) (Hsieh et al., 2010), with the root mean square deviations of 2.89 Å (202 monomer Cα atoms) to PPL2 and 3.48 Å (217 monomer Cα atoms) to ChiNCTU2. Chi23 comprises 14 α-helices and 10 β-sheets (Figure 5B). The catalytic ^113^DxDxE^117^ motif is located in the loop between β6 and α5, and a hydrogen bond is formed between the catalytic residues Asp115 and Glu117.

**TABLE 4:** Diffraction data and refinement statistics of the wild-type Chi23.

**Parameters**	**Chi23**
**Diffraction data**	
Space group	*P*12_1_
**Unit cell**	
a, b, c (Å)	68.62, 87.49, 88.84
α, β, γ (°)	90.00, 95.30, 90.00
Resolution range (Å)	50.00–1.80 (1.86–1.80)^a^
Redundancy	6.4 (6.5)
Completeness (%)	99.9 (99.9)
*R*_merge_ (%)^b^	12.8 (31.6)
*I*/σ*I*	30.3 (10.2)
**Refinement statistics**	
R-factor (%)	21.5
Free R-factor (%)	23.7
**RMSD from ideal geometry**	
Bond lengths (Å)	0.011
Bond angles (°)	1.00
**Ramachandran plot (%)**	
Favored	97.70
Allowed	2.30
Outliers	0
Overall B-factors (Å^2^)	18.51

*^*a*^Numbers in parentheses refer to data in the highest resolution shell. ^*b*^*R*_*merge*_ = Σ_*hkl*_Σ_*i*_| *I*(*hkl*)_*i*_ - < *I*(*hkl*) > | /Σ_*hkl*_Σ_*i*_ < *I*(*hkl*)_*i*_ >.*

Although Chi23 has similar overall structure to the catalytic domains of the other chitinases from the GH18 subfamily B, some differences in their structures are also observed. All known bacterial chitinases from the GH18 subfamily B contain a small β-hairpin subdomain composed of two antiparallel β-sheets (β7 and β8 in ChiNCTU2) and an extra α-helix (α5 in ChiNCTU2) to extend their substrate-binding clefts (Hsieh et al., 2010; Payne et al., 2012; Malecki et al., 2013), which is absent from all eukaryotic chitinases of this subfamily (Terwisscha van Scheltinga et al., 1994; Cavada et al., 2006; Hurtado-Guerrero and van Aalten, 2007; Schuttelkopf et al., 2010; Masuda et al., 2015). For Chi23, it only has such an α-helix, and lacks the β-sheets for the formation of a β-hairpin subdomain (Figures 5C,D). Therefore, different from other bacterial chitinases from the GH18 subfamily B, Chi23 does not have a β-hairpin subdomain. All chitinases of the subfamily B, except for PPL2, harbor several long flexible loops in different regions. For example, three long loops between β4-α4, α7-α8, and α10-β10 are present in ChiNCTU2 to elongate its binding cleft (Hsieh et al., 2010). However, similar to PPL2 (Cavada et al., 2006), Chi23 only has extremely short loops in these corresponding regions (Figures 5C,D). Moreover, surface residues Phe30-Ser44 in Chi23 form two antiparallel β-sheets, which are conserved in nearly all eukaryotic chitinases of the GH18 subfamily B, but not in the bacterial chitinases of this subfamily where only one or no corresponding β-sheet is formed. Taken together, the structure of Chi23 is distinct from those of the other modular endochitinases from the GH18 subfamily B.

### Key Residues Involved in Substrate Binding and Catalysis in Chi23

**FIGURE 6:**
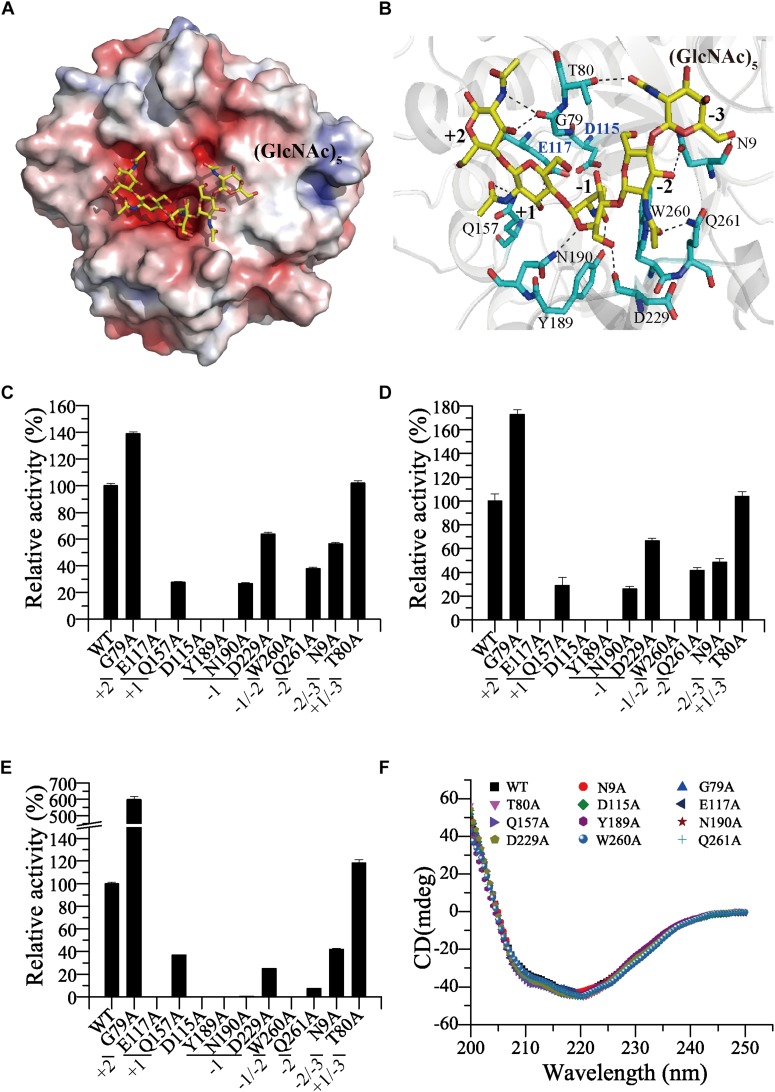
Analyses of the key amino acid residues in Chi23 for substrate binding and catalysis. **(A)** Electrostatic surface view of Chi23 docked with (GlcNAc)_5_ substrate. The positively charged regions are shown in blue and the negatively charged regions in red. **(B)** Detailed structure of Chi23 modeled with (GlcNAc)_5_. Residues involved in the binding and catalysis of (GlcNAc)_5_ are shown as cyan sticks, and the substrate (GlcNAc)_5_ is shown as yellow sticks. Interactions between Chi23 residues and (GlcNAc)_5_ within hydrogen-bond distance are shown as dashed lines. **(C)** Activities of the wild-type Chi23 and its mutants against crystalline chitin. **(D)** Activities of Chi23 and its mutants against colloidal chitin. **(E)** Activities of Chi23 and its mutants against 4-MU-(GlcNAc)_2_. **(F)** CD spectra of Chi23 and its mutants. In **(C–E)**, the activity of the wild-type Chi23 is defined as 100%, and the Chi23 subsites to which the modeled (GlcNAc)_5_ binds are also indicated.

To investigate the substrate-binding mode of Chi23, we tried to obtain the structure of Chi23 binding a substrate. We cocrystallized the wild-type Chi23 and its inactive mutants D115A and E117A with different chitooligomers. Unfortunately, no electron density was observed for the substrate in the structure of Chi23 or its mutants. Instead, we modeled the structure of the wild-type Chi23 in complex with (GlcNAc)_5_ by molecular docking. Several candidates were obtained and we chose the lowest energy candidate for the following analysis. In the modeled structure, (GlcNAc)_5_ is bound in the negatively charged substrate cleft (Figure 6A).

Five sugar subsites from −3 to +2 are revealed in the substrate-binding cleft of the structure of Chi23 docked with (GlcNAc)_5_, and the scissile glycosidic bond between the −1 and the +1 subsites is located close to the side chain of the predicted catalytic Glu117 (Figure 6B). The GlcNAc residue bound at the −1 subsite is in an unfavorable boat conformation, and stabilized by the side chains of Tyr189, Asn190 and Asp229 through hydrogen bonds and by Trp260 through hydrophobic stacking. The boat conformation of the sugar at the −1 subsite is critical for the initiation of the enzymatic cleavage (Brameld and Goddard, 1998). Except for Asp229, other residues involved in the binding of the -1 sugar are highly conserved in the GH18 subfamily B (Figure 2). All the acetamido groups of (GlcNAc)_5_ are stabilized by hydrogen bonds. Residues Thr80, Trp260 and Gln261, Tyr189, Gln157 and Gly79 are hydrogen bonded to the C2-acetamido group of GlcNAc unit at subsites from −3 to +2, respectively, mainly through their side chains. The O3 hydroxyl groups of the −2, +1 and +2 sugar residues also form hydrogen bonds with Asn9, Glu117 and Gly79, respectively. There is also a hydrogen bond between the O6 hydroxyl group of the −3 sugar and the main-chain oxygen atom of Asn9.

To analyze the roles of the potential residues involved in substrate binding and catalysis in chitin degradation, single-point mutations of the residues to Ala were performed in Chi23, and the activities and kinetic parameters of the mutants were measured and compared to those of the wild-type enzyme (Figures 6C–E and Table 5). Mutations of residues Asp115 and Glu117 in the catalytic ^113^DxDxE^117^ motif led to complete loss of enzymatic activity toward insoluble and soluble chitin, demonstrating their key roles in the catalysis. Except for T80A at the −3 subsite and G79A at the +2 subsite, all mutations on the residues predicted to be involved in substrate binding at subsites from −2 to +1 severely decreased or even abolished the activity of Chi23 against crystalline chitin, colloidal chitin or 4-MU-(GlcNAc)_2_, and reduced both the substrate affinity and *k*_*cat*_ of Chi23, indicating that residues Gln157, Tyr189, Asn190, Asp229, Trp260, Gln261 and Asn9 at subsites from −2 to +1 play important roles in Chi23 for the binding and degradation of insoluble and soluble chitin. Mutation T80A had little effect on the enzymatic activity or the *k*_*cat*_/*K*_*m*_ value of Chi23, suggesting that Thr80 possibly contributes little to the binding of soluble and insoluble chitin. Mutation G79A significantly stimulated 4-MU-(GlcNAc)_2_ hydrolysis of Chi23 but had a minor impact on crystalline and colloidal chitin hydrolysis, and similar cases were observed in *Vibrio carchariae* chitinase A (Suginta et al., 2007) and *Bacillus circulans* chitinase ChiA1 (Watanabe et al., 2003), suggesting that the binding and degradation of long-chain chitin tends to be influenced by a cluster of the surface-exposed residues in the substrate cleft rather than by a particular residue. In *Bacillus circulans* chitinase ChiA1, the increase in the hydrolytic activity against chitooligomers by mutations W164A and W285A at subsites +1 and +2, respectively, was perhaps attributed to the weakness of substrate inhibition (Watanabe et al., 2003). However, no inhibitory effect on the Chi23 activity was observed for 4-MU-(GlcNAc)_2_ at the tested concentrations ranging from 0.01 to 0.6 mM, implying that other reason may explain its enhanced activity toward 4-MU-(GlcNAc)_2_, which needs further study. Mutation G79A influenced only the *k*_*cat*_ of Chi23 in 4-MU-(GlcNAc)_2_ degradation but not its *K*_*m*_, consistent with the main chain of Gly79 involved in the substrate binding. CD spectroscopy analysis showed that the secondary structures of the mutants exhibited little deviation from that of the wild-type Chi23, indicating that the changes in the enzymatic activity and kinetic parameters of the mutants are caused by residue substitution rather than structural changes in Chi23 (Figure 6F).

**TABLE 5:** Kinetic parameters of Chi23 and its mutants^a^.

**Enzyme**	***K*_m_**	***V*_*max*_**	***k*_cat_**	**Relative**	***T*_*opt*_**
	**(μM)**	**(nmol/min/mg)**	**(s^–1^ × 10^–3^)**	***k*_cat_/*K*_m_^b^**	**(°C)**
WT-Chi23	64.6 ± 11.7	10.7 ± 0.9	5.4 ± 0.5	100%	60
**Catalytic residues**					
D115A	LD^c^	LD	LD	LD	LD
E117A	LD	LD	LD	LD	LD
**Potential binding residues**					
N9A	90.1 ± 13.4	4.2 ± 0.3	2.1 ± 0.2	28.1%	60
G79A	72.6 ± 6.9	57.6 ± 2.5	29.5 ± 1.3	477.3%	60
T80A	85.6 ± 7.2	14.4 ± 0.5	7.3 ± 0.3	101.6%	60
Q157A	104.1 ± 17.5	4.4 ± 0.3	2.2 ± 0.2	25.5%	60
Y189A	LD	LD	LD	LD	LD
N190A	77.4 ± 16.2	0.04 ± 0.004	0.02 ± 0.002	0.3%	60
D229A	80.1 ± 6.3	2.7 ± 0.1	1.4 ± 0.05	20.4%	45
W260A	LD	LD	LD	LD	LD
Q261A	86.3 ± 6.6	0.9 ± 0.04	0.5 ± 0.02	6.3%	50

*^*a*^Reactions were conducted in triplicate in McIlvaine’s buffer (pH 5.0) at 60°C using 4-MU-(GlcNAc)_2_ as substrate over a concentration range of 0.01–0.6 mM. ^*b*^Percentages were calculated relative to the wild-type Chi23. ^*c*^LD indicates that the value was less than the limit of detection.*

### Comparison of the Substrate-Binding Clefts of Chi23 and Other GH18 Endochitinases

**FIGURE 7:**
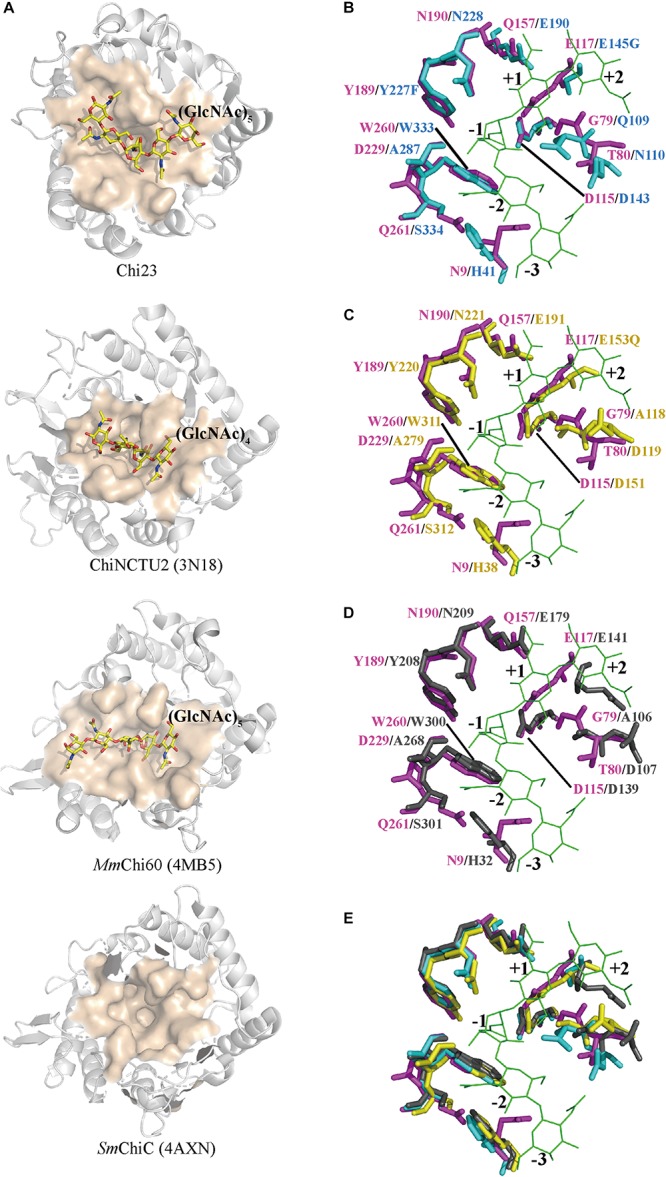
Structural comparison of the substrate-binding clefts of Chi23, ChiNCTU2, *Mm*Chi60 and *Sm*ChiC. **(A)** The substrate-binding clefts of Chi23, ChiNCTU2 (PDB code 3N18), *Mm*Chi60 (PDB code 4MB5) and *Sm*ChiC (PDB code 4AXN). The substrate-binding clefts are shown as surface and colored in apricot. The (GlcNAc)_5_ docked into Chi23, the (GlcNAc)_4_ bound to ChiNCTU2, and the (GlcNAc)_5_ bound to *Mm*Chi60 are shown as yellow sticks. **(B–E)** Superposition of the substrate-binding clefts of Chi23 (magenta) and ChiNCTU2 (cyan) or/and *Mm*Chi60 (yellow) or/and *Sm*ChiC (gray). In **(B–E)**, the (GlcNAc)_5_ docked into Chi23 is shown as green lines. Residues involved in the catalysis and substrate binding are shown as sticks, and colored in magenta for Chi23, in cyan for ChiNCTU2, in yellow for *Mm*Chi60, and in gray for *Sm*ChiC.

In the GH18 family, several endochitinases active on crystalline chitin with specific activities ranging from 0.03 to 1.5 U/mg have been reported, including *Mm*Chi60, *Cj*Chi18C, *Sm*ChiC, *Sp*ChiC and *Ss*Chi18B, which are all modular enzymes containing one or more CBDs in addition to the CaD (Suzuki et al., 1999; Stefanidi and Vorgias, 2008; Purushotham et al., 2012; Monge et al., 2018; Sun et al., 2019). Compared to other GH18 endochitinases, Chi23 lacks a CBD, but has similar efficiency on crystalline chitin degradation (Table 2). To further reveal the mechanism for chitin degradation, especially for crystalline chitin, by Chi23, a comparative analysis of the structures of Chi23 and other GH18 endochitinases was carried out (Figure 7).

Similar to the catalytic domains of other GH18 endochitinases, Chi23 has a shallow groove-like binding cleft (Figure 7A). However, Chi23 displays differences in substrate binding involving residues Asn9, Asp229 and Gln261 compared to other GH18 endochitinases (Figures 7B–E). The side chain of Asp229 in Chi23 is hydrogen bonded to the O6 group of the -1 sugar to keep its boat conformation, which is replaced by a small hydrophobic Ala conserved in all other chitinases of the GH18 subfamily B (Figures 7B–E). However, mutant D229A of Chi23 showed significantly reduced activity to crystalline and colloidal chitin and a much lower activity to 4-MU-(GlcNAc)_2_ (Figures 6C–E), demonstrating the importance of Asp229 in Chi23 for the hydrolysis of insoluble and soluble chitin. Gln261 in Chi23 stabilizes the acetamido group of the −2 sugar through its side chain, and mutation analysis indicates that Gln261 is important for both insoluble and soluble chitin degradation (Figures 6B–E). The counterpart of Gln261 of Chi23 is a small hydrophilic Ser that is conserved in most reported chitinases of the GH18 subfamily B (Figures 7B–E). No interaction is found between the corresponding Ser and bound chitooligomers in ChiNCTU2 (Hsieh et al., 2010) or *Mm*Chi60 (Malecki et al., 2013). Asn9 participates in the substrate binding of Chi23 through either its side chain or main chain. Asn9 of Chi23 is replaced by a conserved aromatic His in other bacterial chitinases or by a small conserved Gly in eukaryotic chitinases in the GH18 subfamily B. Mutation analysis indicates that Asn9 in Chi23 plays an important role in insoluble and soluble chitin degradation (Figures 6C–E). However, its counterparts in both ChiNCTU2 (His41) (Hsieh et al., 2010) and *Mm*Chi60 (His38) (Malecki et al., 2013) are far away from the bound chitooligomers, and only a weak hydrophobic stacking interaction is formed between His38 of *Mm*Chi60 and chitopentose, and no interaction forms between His41 of ChiNCTU2 and chitooligomers due to the buried property of this residue. Altogether, these data indicate that the three unique residues Asn9, Asp229 and Gln261 play an important role in Chi23 for chitin degradation, and their cumulative roles in substrate binding and catalysis contribute to the activity of the single-domain Chi23 against both crystalline and soluble chitin.

### Structural Basis for the High Thermostability of Chi23

**FIGURE 8:**
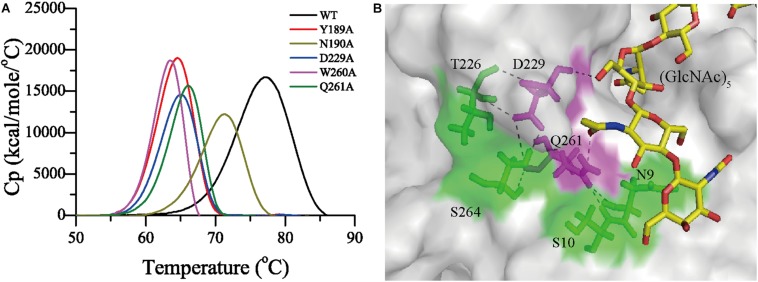
Residues involved in the thermostability of Chi23. **(A)** Differential scanning calorimetry (DSC) thermograms for Chi23 and its mutants. **(B)** The hydrogen bond networks in Chi23 involving residues Asp229 and Gln261 (magenta sticks with hydrogen atoms shown). (GlcNAc)_5_ docked into Chi23 is shown as yellow sticks.

To study the contribution of identified substrate-binding residues to the thermostability of Chi23, the apparent melting temperatures (*T*_*m*_) and optimal temperatures (*T*_*opt*_) of Chi23 and its mutants were measured and compared. The *T*_*m*_ and *T*_*opt*_ values of mutants N9A, G79A, T80A and Q157A were similar to that of Chi23 (a *T*_*opt*_ of 60°C and a *T*_*m*_ of 77°C), suggesting that these residues may contribute little to the thermostability of Chi23. In contrast, mutation Q261A at the −2 subsite and all mutations including Y189A, N190A, D229A and W260A at the −1 subsite led to 6–14°C reduction in the *T*_*m*_ of Chi23 (Figure 8A), indicating the important roles of these substrate-binding residues at the −1 and −2 subsites in stabilizing the protein structure of Chi23. Among these five mutants, the *T*_*opt*_ values of mutants Y189A and W260A are undetectable due to the complete loss of their activity, mutant N190A had an unchanged *T*_*opt*_, and mutants D229A (45°C) and Q261A (50°C) had much lower *T*_*opt*_ values than the wild-type Chi23 (60°C) (Table 5). Moreover, both D229A and Q261A became very unstable at 60°C, further supporting that residues Asp229 and Gln261 play an important role in maintaining the structural stability of Chi23. A detailed structural analysis of Chi23 showed that the side chain of Asp229 forms four hydrogen bonds with the solvent-exposed residues Thr226 and Ser264, and that the side chain of Gln261 is hydrogen bonded to residues Ser10 and Asn9 lining on the substrate cleft surface (Figure 8B). The mutation of the hydrophilic Asp229 or Gln261 to small hydrophobic Ala led to the disruption of the hydrogen bond networks involving these two residues and their surrounding residues in Chi23, and therefore the protein structure of Chi23 became less rigid and less stable at high temperatures. The contribution of hydrogen bonds to protein thermostability was also reported in other glycoside hydrolases (Vogt et al., 1997; Han et al., 2018). In addition, the absence of long flexible loops may also benefit the high thermostability of Chi23.

### Chi23 and Its Homologs Form a New Group of GH18 Endochitinases

**FIGURE 9:**
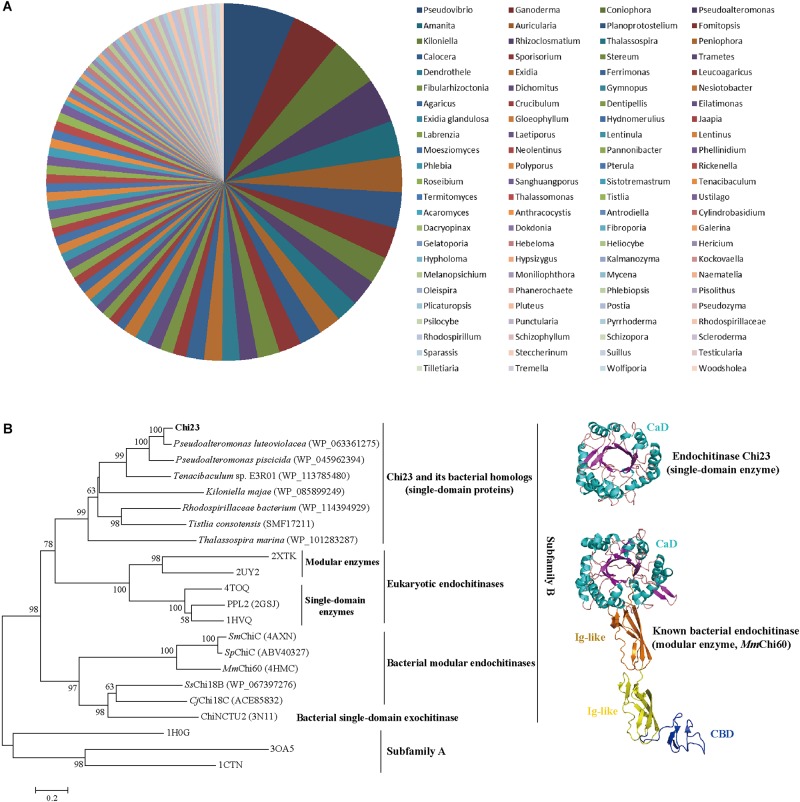
Distribution in bacteria **(A)** and phylogenetic analysis **(B)** of Chi23 and its homologs. **(A)** Distribution of Chi23 homologs in bacteria from marine and soil environments. All the Chi23 homologs are potential single-domain proteins. **(B)** A neighbor-joining tree of the GH18 subfamily B endochitinases. Bootstrap analysis of 1,000 replicates was conducted, and values above 50% are shown. Chitinases from the GH18 subfamily A were used as an outgroup. The crystal structures of Chi23 and *Mm*Chi60 are also shown.

Among the reported GH18 endochitinases, Chi23 is most closely related to the eukaryotic single-domain endochitinase PPL2 (Cavada et al., 2006), with a low sequence identity of 30%, suggesting that Chi23 is a new member of the GH18 endochitinases. In addition to sequence, Chi23 is also different from reported GH18 bacterial endochitinases in structure due to the lack of the CBD and the β-hairpin subdomain (Figure 5). Blasting analysis revealed that more than 140 homologs of Chi23 are found in the NCBI non-redundant (nr) protein database, which are all potential single-domain proteins and distributed in various bacterial species (Figure 9A). Phylogenetic analysis showed that Chi23 and its homologs are clustered as a group separate from the cluster of the reported GH18 endochitinases (Figure 9B). Chi23 and its homologs are more closely related to GH18 eukaryotic endochitinases than to reported GH18 bacterial modular endochitinases (Figure 9B). Thus, based on the differences in sequence and structure between Chi23 (and its homologs) and other GH18 bacterial endochitinases, Chi23 and its homologs are suggested to represent a new group of GH18 endochitinases.

## Conclusion

Chi23 is a marine bacterial endochitinase capable of degrading crystalline and colloidal chitin. Chi23 and its homologs are widespread in bacteria and represent a new group of GH18 endochitinases, suggesting that they may play an important role in marine and terrestrial chitin degradation and recycling. Chi23 is also a thermostable enzyme, thus making it a potential candidate for the industrial processing of chitin. Our structure-function analysis on Chi23 broadens our knowledge on the molecular mechanisms for the GH18 endochitinases in chitin degradation, and offers a basis for developing the industrial applications of Chi23 and other GH18 chitinases.

## Data Availability Statement

The nucleotide sequence encoding Chi23 has been deposited in the GenBank database with the accession number MK948094. The structure of wild-type Chi23 has been deposited in PDB under the accession number 6K7Z.

## Author Contributions

Y-ZZ and X-YS designed the research. P-YL and X-LC directed the research. Y-JW, W-XJ, YZ, and Y-SZ performed the experiments. P-YL solved the structure. H-YC, C-YL, and PW helped in analyzing the structural data. Y-JW, P-YL and X-LC wrote the manuscript.

## Conflict of Interest

The authors declare that the research was conducted in the absence of any commercial or financial relationships that could be construed as a potential conflict of interest.

## Footnotes

1 https://www.schrodinger.com/
